# Supplementary material for: Complexome profiling of the Chlamydomonas psb28 mutant reveals TEF5 as an early PSII assembly factor
Source: Plant Cell. 2025 Mar 17;37(6):koaf055. doi: 10.1093/plcell/koaf055 (PMC12164590; doi:10.1093/plcell/koaf055)
Supplement: koaf055_Supplementary_Data [file koaf055_supplementary_data.zip › psb28_interactive.zip!/plots/intensity/qualityPlots/peptides/_Cre11.g467755.t1.1.html]

### Cre11.g467755.t1.1

Trivial name:   
SeqCoverage [median]: 11.30  
Mass: 31.67 kDa  
  
 

| Sequence | MissedCleavage | Mass [Da] | wtIntensity | muIntensity | Score |
| --- | --- | --- | --- | --- | --- |
| DKVVEIVASPSAPR | 1 | 1466.81 | 0.00 | 643.40 | 41.38 |
| EDSLFGLDSDPGR | 0 | 1406.63 | 295.24 | 0.00 | 75.29 |
| LVDAASR | 0 | 730.40 | 1008.84 | 1455.47 | 91.63 |
| REDSLFGLDSDPGR | 1 | 1562.73 | 0.00 | 3044.47 | 53.40 |
| VVEIVASPSAPR | 0 | 1223.69 | 0.00 | 2452.19 | 128.03 |
|  |  |  | 1304.1 | 7595.5 |  |
